# Anti-Inflammatory Effect of Protopine through MAPK and NF-κB Signaling Regulation in HepG2 Cell

**DOI:** 10.3390/molecules27144601

**Published:** 2022-07-19

**Authors:** MinGyu Kim, Hyuck Kim, Hojun Kim

**Affiliations:** Department of Rehabilitation Medicine of Korean Medicine, Dongguk University, 814 Siksa-dong, Ilsandong-gu, Goyang-si 10326, Korea; kmk63819@gmail.com

**Keywords:** HepG2, PMA, protopine

## Abstract

Protopine is a substance used for hemostasis with an anti-inflammatory action and is one of the substances that are actively undergoing experiments to confirm their utility as anticancer agents. This study examined the molecular changes in the cellular signaling pathways associated with inflammatory responses in phorbol 12-myristate 13 acetate (PMA)-induced human hepatocellular carcinoma cell line (Hep G2). The inhibition of PMA-induced phosphorylation of I-κB in HepG2, the effect of protopine on the MAPK signals, the inhibition of COX-2 activity, and the inhibition of MMP-9 as a medium of inflammatory response were evaluated by Western blot and qPCR. The effect of protopine on the survival rates in HepG2 cells was evaluated and found to be stable to a processing concentration of up to 40μM. Subsequent Western blot analyses showed that protopine blocks the transfer of the MAPKs cell signals induced by PMA and the transfer of the subunit of the nuclear factor-kappa B (NF-κB) to the nucleolus. Protopine inhibited the kappa alpha (I-κBα) phosphorylation in the cytosol and blocked PMA-induced inflammation via COX-2 activity inhibition. The expression of MMP-9 at the gene and protein levels, which is associated with cell migration and metastasis, was reduced by protopine.

## 1. Introduction

Protopine is an alkaloid component contained mainly in Papaveraceae plants, and it is known for its excellent anti-inflammatory action as a representative biological effect [1,2,3,4,5,6,7]. Garcia-Gil (2021) and Son (2019) reported that protopine induces apoptosis in colon cancer cells and prostate cancer cells and has analgesic action by inhibiting the histamine H1 receptor and platelet aggregation [5,6]. The hepatoprotective activity and anti-inflammatory effects were also reported [2,7]. Protopine was reported to attenuate inflammatory symptoms by inhibiting the mitogen-activated protein kinase (MAPK)/nuclear factor kappa B (NF-κB) signaling pathway in Raw264.7 cells [2]. Based on these results, this study shows that protopine inhibits hepatitis in PMA-stimulated HepG2 cells by inhibiting the MAPK/IκB/NF-κB signaling pathway. Mitogen-activated protein kinase (MAPK) is composed of extracellular signal-regulated kinases (ERKs), c-Jun NH2-terminal kinases (JNKs), and p38-MAPK (p38), contributing to inflammation, cell survival, and natural cell death [8]. In particular, in human hepatocellular carcinoma cells (HepG2), the signal transduction process is initiated by activating the signal transduction enzyme protein kinase C (PKC) by phorbol 12-myristate 13-acetate (PMA) [9,10,11,12,13,14,15]. Studies have shown that MAPKs contribute to the activation process in which inflammatory stimuli are transmitted to the nucleus of cells through phosphorylation of the inhibitor of nuclear factor kappa B (I-κB), a subunit of NF-κB. In addition, protopine exhibits anti-inflammatory efficacy by regulating the NF-κB signaling factor in various cell lines [9,10]. NF-κB induces the expression of various inflammatory genes, including genes encoding cytokines and chemokines, and participates in inflammatory regulation. In addition, MMP-9, a factor that causes cell migration involved in cell suicide, is also activated. On the other hand, there are insufficient reports on whether the anti-inflammatory effects of protopine regulate the PMA-induced MAPKs activity in human liver cancer cell lines. This study examined the protopine-induced inhibition of PMA-induced IκB phosphorylation in HepG2 cells and its effects on MAPK signaling, the inhibition of cyclooxygenase-2 (COX-2) activity as a mediator of inflammatory responses, and inhibition of matrix metalloproteinase (MMP)-9.

## 2. Results

### 2.1. Effect of Protopine on the HepG2 Cell Viability

An MTT assay was performed to evaluate the effect on the HepG2 cell viability. The concentration-dependent decrease in protopine at a concentration of 0–50 μM. For the evaluation of cell viability, the maximum concentration of protopine was set to 40 μM with low cytotoxicity, and the experiments were performed at concentrations of 10 μM, 20 μM, and 40 μM in the cell experiments (Figure S1).

### 2.2. Inhibitory Effect of Protopine on the PMA-Induced Inflammatory Transcription Factor NF-κB Signals in HepG2 Cells

**Figure 1:**
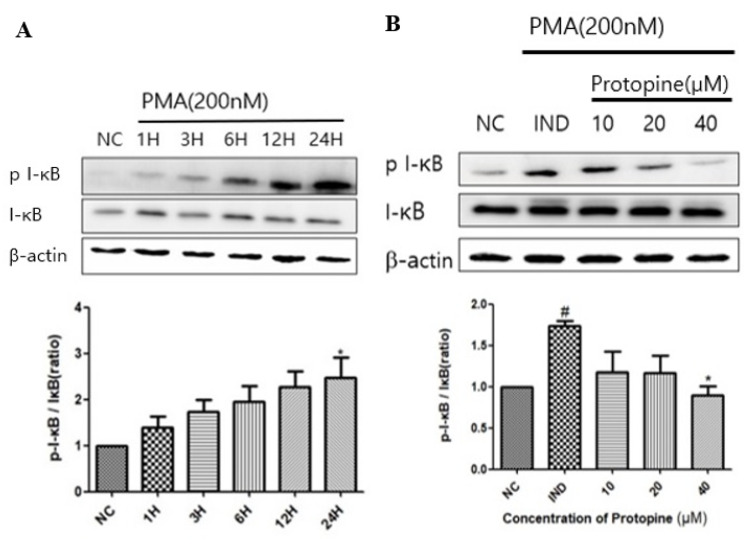
Protopine regulated I-κBα translocation in PMA-stimulated HepG2 cells. (**A**) Western blot analysis of time-dependent I-κBα phosphorylation after treatment with PMA 200 nM in HepG2 cells. (**B**) Cells were pretreated with protopine 40 μM for 1 h and then treated with PMA 200 nM for 24 h. The level of I-κBα protein phosphorylation was determined by Western blot. The results are presented as the means ± SD. # *p* < 0.05 indicates significantly different from the normal control. * *p* < 0.05 indicate a significant difference from the PMA group. NC, normal control.

**Figure 2:**
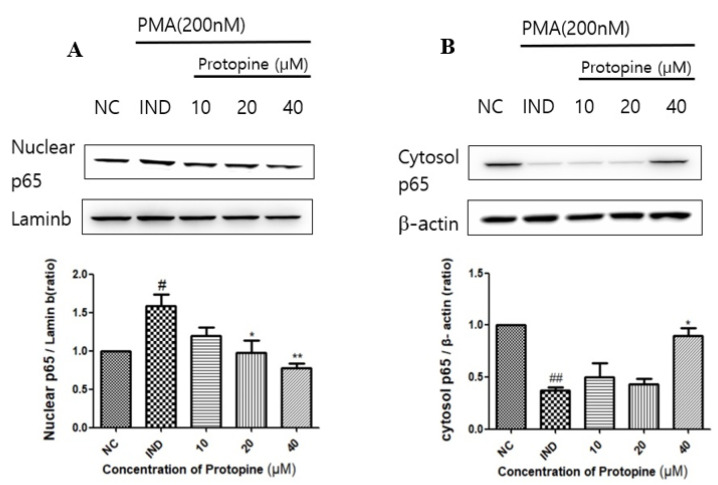
Protopine regulated NF-κB translocation in PMA-stimulated HepG2 cells. (**A**) Protopine treatment induced nuclear NF-κB expression in a concentration-dependent manner. (**B**) Protopine treatment induced cytosol NF-κB expression in a concentration-dependent manner. Results are presented as means ± SD. # *p* < 0.05 and ## *p* < 0.01 indicates significantly different from control group. * *p* < 0.05, ** *p* < 0.01 indicate significantly different from PMA group. NC, normal control group; IND, inducer.

**Figure 3:**
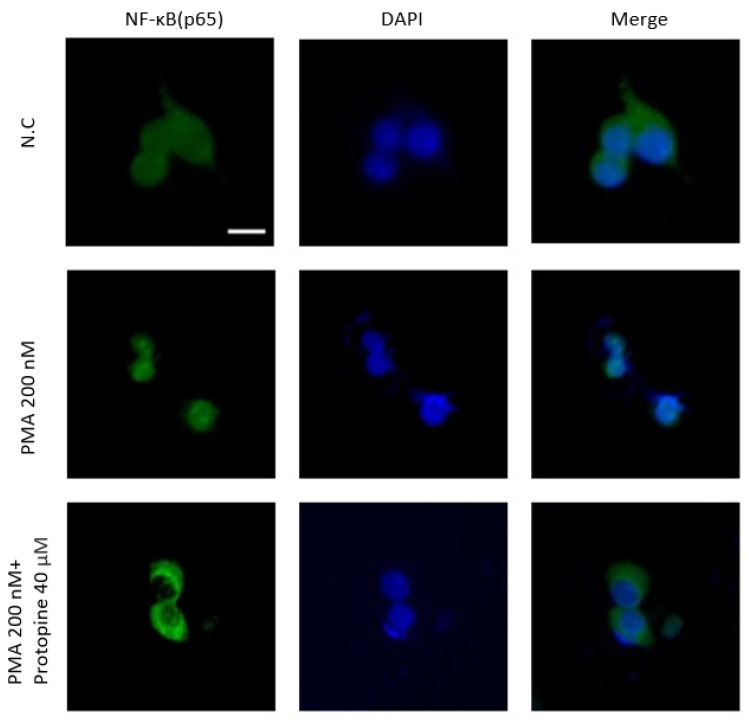
Immunofluorescence images of HepG2 cells that were treated with PMA and protopine and analyzed for NF-κB expression. PMA treatment promoted the translocation of NF-κB protein from the cytoplasm to the nucleus of HepG2 cells. When the cells were treated with 200 nM PMA alone, translocation of the NF-κB protein from the cytoplasm to the nucleus occurred. The protopine treatment inhibited the translocation of NF-κB protein from the cytoplasm to the nucleus in HepG2 cells. Scale bar = 5 μm.

To verify the expression of the NF-κB signaling pathway, transcription factors associated with inflammatory responses induced by PMA, I-κBα, were confirmed by a time-dependent Western blot. As a result, phosphorylation of I-κBα was highest 24 h after the PMA treatment (Figure 1A). In addition, the cells were also treated at different concentrations. When I-κBα was treated with protopine at 40 μM, phosphorylation was reduced compared to the group treated with PMA alone (Figure 1B). After separating the cytoplasm and the nucleolus, the movement from the cytoplasm to the nucleolus was inhibited in a concentration-dependent manner by Western blot (Figure 2A). Immunofluorescence was used to visualize the phosphorylation inhibitory effect. As a result, the nucleus moved out of the cytoplasm when the cells were treated with PMA alone. The nucleus was inhibited from moving out of the cytoplasm when treated with protopine at 40 μM (Figure 3).

### 2.3. Effect of Protopine on the Inhibition of PMA-Induced Expression of COX-2 and MMP-9 in HepG2 Cells

**Figure 4:**
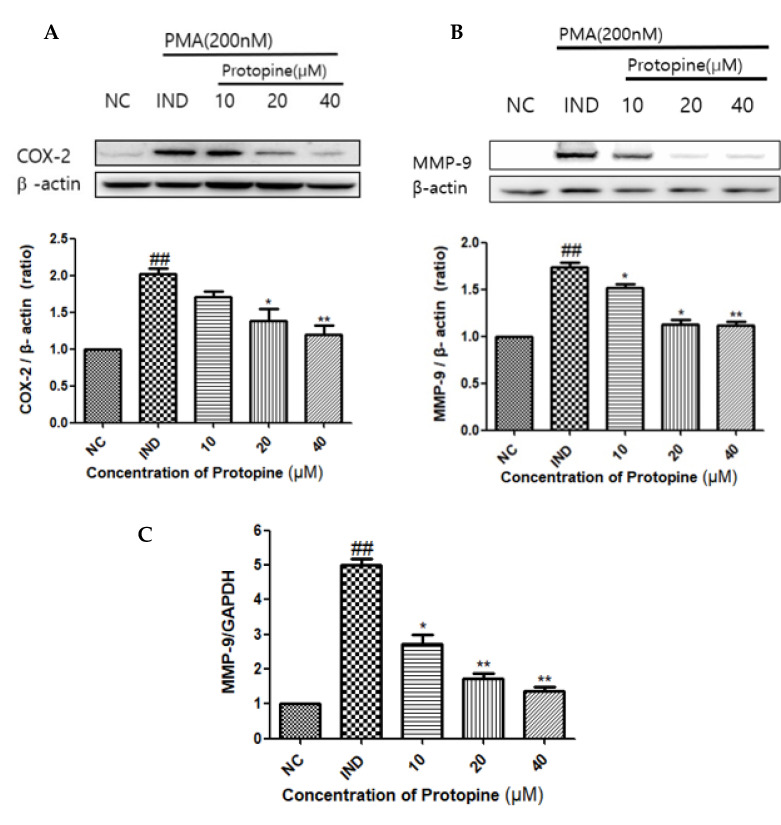
Protopine regulated COX-2 and MMP-9 in PMA-stimulated HepG2 cells. (**A**) COX-2, confirmed by Western blot, increased expression during the PMA treatment and decreased in a concentration-dependent manner upon the protopine treatment. (**B**) MMP-9, confirmed by Western blot, increased the expression when treated with PMA, and showed a high inhibition rate when treated with 40 µM of protopine. (**C**) MMP-9 mRNA levels were determined by RT-PCR analysis. MMP-9 showed the highest inhibition rate even at the mRNA level when treated with 40 µM protopine. Results are presented as the mean ± SD. ## *p* < 0.01, indicating a significant difference from the control. * *p* < 0.05, ** *p* < 0.01, indicating a significant difference from the PMA group. NC, normal control; IND, inducer.

The expression and inhibition of the NF-κB signaling pathway, an inflammatory transcription factor induced through PMA, were confirmed, and Western blot and RT-qPCR were performed. The expression of COX-2 induced by PMA was decreased significantly (*p* < 0.0038) by protopine in a concentration-dependent manner (Figure 4A). Western blot analysis of MMP-9 showed a significantly (*p* < 0.0017) protopine-induced decrease at 40 μM (Figure 4B). RT-qPCR confirmed that the decrease occurred in a concentration-dependent manner and showed a high inhibition rate at 40 μM (Figure 4C).

### 2.4. Effect of Protopine on the PMA-Induced Inflammatory Signaling Pathway MAPK in HepG2 Cells

**Figure 5:**
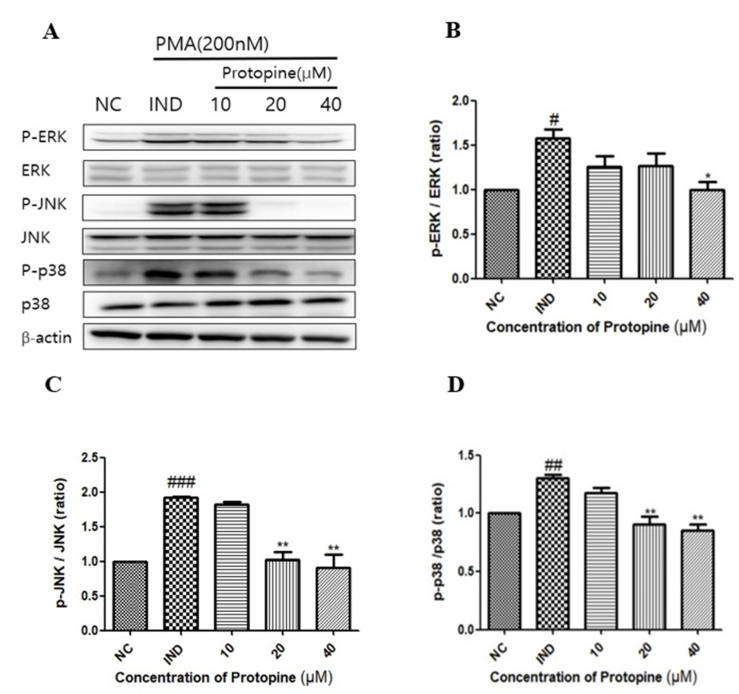
Protopine PMA-stimulated MAPK activity in HepG2 cells. (**A**) Levels of MAPK expression determined by Western blot. (**B**) ERK1/2 activated by PMA inhibited its activation in a concentration-dependent manner on protopine. (**C**) Protopine inhibited JNK activation in a concentration-dependent manner. (**D**) Protopine suppressed the activity of p38 to a high level when treated with 40 µM. The results are presented as mean ± SD. # *p* < 0.05 ## *p* < 0.01, and ### *p* < 0.001 indicate significant differences from the control group. * *p* < 0.05, ** *p* < 0.01, indicating a significant difference from the PMA group.

The expression and inhibition of the NF-κB signaling pathway, an inflammatory transcription factor induced through PMA, were confirmed, and the regulation of the MAPKs signals corresponding to higher levels of signals by protopine was confirmed by Western blot. ERK expression was strongly inhibited by protopine at 40 μM (*p* < 0.0137, Figure 5A,B). On the other hand, JNK and p38 were decreased significantly (*p* < 0.0016) in a concentration-dependent manner and showed a high inhibition rate at 40 μM (Figure 5C,D).

## 3. Discussion

Protopine is an alkaloid found in Papaveraceae with anti-inflammatory effects [2,4]. According to the results of Rathi (2008) and Alam (2019), there are studies on the regulation of MAPKs activity [2,5,6,7]. Combining the above two previous studies, the anti-inflammatory effect of protopine inhibits inflammation by suppressing the changes in MAPK activity, which is also applied to liver cells. Cellular studies have shown that regulation of the MAPK pathway can inhibit the inflammatory response and be a target of the ultimate molecular action point among the branches of the signaling process. The phosphorylation of I-κBα after the MAPK signaling is expected. This has been applied as a basic research model to the study of inflammatory responses in tumor cells, such as HepG2. In this study, the changes in the MAPKs activity were measured using PMA as an inducer in HepG2 cells, a human liver cancer cell line. Therefore, in regulating the PMA-induced phosphorylation of PMA-induced cell signaling molecules, the process is related to MAPK-related inflammatory NF-κB signaling [16,17,18]. NF-κB regulates the pleiotropic regulation of a wide range of pro-inflammatory genes, such as iNOS and COX-2, as well as various cytokines and chemokines. Various studies have reported an increased expression of inflammation in response to NF-κB binding to the gene promoter regions. IκB maintains NF κB in the cytoplasm by preventing nuclear localization [19,20,21]. Various inflammatory stimuli, such as LPS and PMA, induce NF-κB activation through the phosphorylation and subsequent degradation of IκB proteins [22]. The present study indicates that protopine inhibits the PMA-activated phosphorylation of IκB, with time-dependent and concentration-dependent results. Therefore, inhibiting the NF-κB signaling pathway by protopine may lead to the downregulation of pro-inflammatory mediators, leading to anti-inflammatory effects. MAPKs, such as ERK, JNK, and p38, play important roles in regulating PMA-stimulated cytokine and chemokine production. Activation of MAPK through PMA could further stimulate other kinase proteins, followed by the nuclear translocation of NF-κB, which in turn would activate the transcription of pro-inflammatory genes with NF-κB binding sites in their promoters [23,24]. In Western blot analysis, protopine inhibited the transduction of PMA-induced MAPKs cell signaling and blocked the nucleoplasm transport of p65, a subunit of nuclear factor kappa B (NF-κB). In particular, protopine inhibited kappa B alpha (I-κBα) phosphorylation of the cytosol significantly. As a result, the performance of blocking PMA-induced inflammation by inhibiting COX-2 activity was confirmed. Finally, after confirming the expression of MMP-9 involved in cell migration and metastasis, protopine induced a decrease in expression at both the gene and protein levels [25]. The efficacy of MMP-9-activated protopine to block inflammation is related to cellular abnormalities, i.e., excessive cell proliferation and migration, differentiation and invasion, and apoptosis. These results show a similar trend to previous studies [26,27,28]. Unlike protopine, which exhibited various physiological activities in cells, the limitation of this study was the lack of verification using animal experiments. A study of the mechanism of overcoming inflammatory-response-mediated diseases can be a new research goal in the future.

## 4. Materials and Methods

### 4.1. Material

Human hepatocellular carcinoma cells (HepG2) were purchased from the Korean Cell Line Bank (KCLB, no. 88065, Seoul, Korea) and cultured in 10% fetal bovine serum (FBS) (Invitrogen, Grand Island, NY, USA) and penicillin/streptomycin mix (Invitrogen, Grand Island, NY, USA) containing Dulbecco’s Modified Eagles Medium (DMEM) (Hyclone, Logan, UT, USA). Protopine was purchased from ChemFaces and cell experiments (Figure 1). Phorbol 12-myristate 13-acetate (PMA) was supplied by Sigma Chemicals (St. Louis, MO, USA) and used. The antibodies used in Western blot analysis, JNK, p-JNK, p38, p-p38, NF-κB, I-κBα, pI-κBα, ERK, p-ERK, MMP-9, COX-2, and β-actin, were obtained from Cell Signaling Technology (Beverly, MA, USA). Anti-mouse and anti-rabbit (Santa Cruz Biotechnology, Dallas, TX, USA) were used for the secondary antibody.

### 4.2. Cell Culture and Viability Measurement

HepG2 cells were cultured at 37 °C and 5% CO_2_ using Dulbecco’s Modified Eagles Medium (DMEM) containing 10% FBS and the penicillin/streptomycin mix. A MTT assay was performed to evaluate the effect of protopine on cell survival. First, after seeding cells at 2 × 10⁵ cells/mL in 96 wells, the cells were incubated for 24 h. The supernatant was removed, and DMEM (Dulbecco’s Modified Eagles Medium) without FBS was added. The cells were then incubated for 24 h again. Subsequently, 5 mg/mL of MTT was added to the medium at a ratio of 1:9 and incubated at 37 °C in dark conditions for two hours. After removing the supernatant and drying, the formazan was dissolved in DMSO (Dimethyl Sufoxide), and the absorbance was measured at 540 nm (VersaMax, Molecular Device, San Jose, CA, USA).

### 4.3. Western Blot Analysis

The protopine-induced PMA-induced inflammatory signaling process was examined. HepG2 cells were seeded into six-well plates at 2 × 10⁵ cells/mL. After 24 h, the medium was exchanged for DMEM not containing FBS, treated with 200 nM PMA, and then with protopine (10, 20, or 40 μM) after one hour. The proteins were harvested from the cells after 24 h of incubation. Protein quantification was performed using a BCA protein assay kit (Thermo Fisher Scientific, Waltham, MA, USA). The absorbance was measured at 562 nm, and the protein value was quantified by substituting it into a standard curve. The protein was quantified at 20 μg/lane and running and transferred for 10% sodium dodecyl sulfate (SDS)-polyacrylamide gel electrophoresis (PAGE). For the blocking procedure to prevent the binding of non-specific antibodies, the membrane was reacted with Tris-buffered saline-Tween 20 (TBS-T) containing 5% bovine serum albumin (BSA) for one hour and using TBS-T. The membrane was washed three times for five minutes each. The primary antibody was prepared in a ratio of 1:1000 in TBS-T containing 3% BSA and reacted at 4 °C for 12 h. After overnight incubation, the membranes were washed three times with TBS-T for five minutes each. The secondary antibody containing 1% BSA was reacted with TBS-T at a ratio of 1:3000 at room temperature for two hours. Finally, immunoreactive fluorescence was induced using an enhanced chemiluminescent (ECL) solution (Super Signal West Pico, Thermo Fisher Scientific, Waltham, MA, USA), and analyzed on a Western blot imaging system (Fusion solo, Marne-la-Vallee, France). For the Western blot band images, the density of the band was measured using Image J 1.52a software, and a normalization process was performed using the housekeeping gene β-actin and a protein suitable for each experiment (Table S1).

### 4.4. Quantitative Polymerase Chain Reaction

A real-time polymerase chain reaction (qPCR) was performed to evaluate the effects of protopine on PMA-induced gene expression of MMP-9. The HepG2 cells were inoculated at 2 × 10^5^ cells/mL in a 60 mm cell culture vessel and cultured for 24 h. The cells were treated with protopine (10, 20, or 40 μM) and 200 nM PMA and incubated in DMEM without FBS for 24 h. The total RNA was isolated using TRIzol reagent (Thermo, Waltham, MA, USA). The isolated RNA sample was quantified at 260 nm and 280 nm and synthesized as cDNA according to the manufacturer’s manual provided using AccuPower RT PreMix (Bioneer, Daejeon, Korea) and Oligo (dt) 18 primer (Invitrogen, Carlsbad, CA, USA). In a polymerase chain reaction (PCR), 10 μL of 2X SYBR green Master Mix (Bioneer, Seoul, Korea), 8 μL of ultrapure water (Bioneer, Seoul, Korea), and 10 pmol/μL primers (Macrogen, Seoul, Korea) were mixed sequentially for cDNA in the polymerase chain reaction (PCR) Light Cycler 480. The amplification process was performed in a PCR System (Roche, Basel, Switzerland). The stepwise DNA denaturation, primer annealing, and extension reaction were polymerized in 45 cycles (Table S2).

### 4.5. Immunofluorescence

Immunofluorescence staining was performed to confirm the effect of protopine on blocking PMA-induced NF-κB (p65) migration from the cytoplasm to the nucleus. First, HepG2 cells were grown after seeding on Lab-Tek II chamber slides (Nalgene Nunc, Springfield, IL, USA) at 2 × 10^3^ cells/mL, fixed at room temperature for 10 min using 4% formaldehyde, and then on a 0.1% Triton X-100 induced cell membrane permeabilization at room temperature for 10 min. Subsequently, after blocking using 1% BSA for one hour, the NF-κB(p65) primary antibody was diluted in 1% BSA at 2 μg/mL to induce binding overnight. Subsequently, the fluorescence-treated secondary antibody (Invitrogen, Grand Island, NY, USA) was diluted in 1% BSA at 2 μg/mL, reacted at room temperature for one hour, and observed through a fluorescence microscope (BX50, Olympus, Tokyo, Japan).

### 4.6. Statistical Analysis

Statistical analysis of all experimental results was expressed as the mean ± standard deviation (means ± SD), and one-way ANOVA was performed using the GraphPad Prism 5 (GraphPad Software, San Diego, CA, USA) program to determine the statistical significance of each group. Significance was accepted only when *p* < 0.05, *p* < 0.01, and *p* < 0.001 compared with Tukey’s multiple comparison test.

## 5. Conclusions

This study examined the effects of protopine on the viability of HepG2 cells. The process was stable up to a treatment concentration of 40 μM. Western blot analysis showed that protopine inhibited the transduction of the PMA-induced MAPKs cell signal and blocked the nucleoplasm movement of p65, a subunit of nuclear factor kappa B (NF-κB). Protopine inhibited kappa B alpha (I-κBα) phosphorylation in the cytosol and blocked PMA-induced inflammation by inhibiting COX-2 activity. Finally, after confirming the expression of MMP-9 in cell migration and metastasis, protopine induced a decrease in expression at both the gene and protein levels. Overall, protopine helped to regulate the inflammatory response induced by PMA. Since MMP-9 in the protein and mRNA levels was reduced by the protopine, it may be worthwhile to study the effect of this compound on cell migration and cell suicide in future studies.

## Data Availability

Not applicable.

## Supplementary Materials

The following supporting information can be downloaded at: https://www.mdpi.com/article/10.3390/molecules27144601/s1, Figure S1. Effect of protopine on HepG2 cell viability, Table S1. Antibody used for Western blot analysis, Table S2. PCR primers used for quantitative real-time PCR.
