# Supplementary material for: Anti-Inflammatory Effect of Protopine through MAPK and NF-κB Signaling Regulation in HepG2 Cell
Source: Molecules. 2022 Jul 19;27(14):4601. doi: 10.3390/molecules27144601 (PMC9324321; doi:10.3390/molecules27144601)
Supplement: Supplementary file 1 [file molecules-27-04601-s001.zip › molecules-1780780-supplementary.pdf]

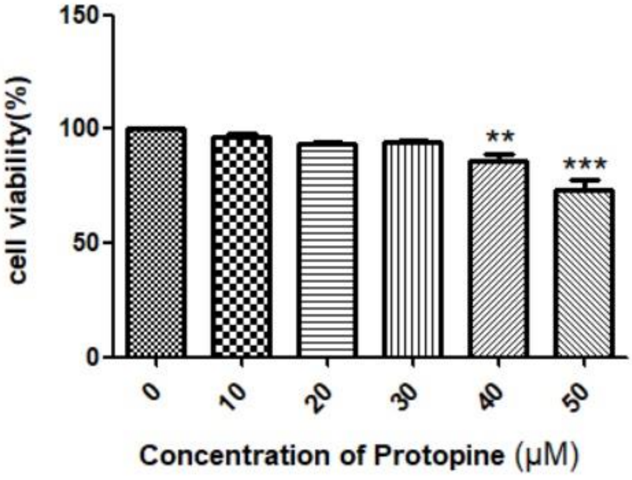

Figure S1. Effect of protopine on HepG2 cell viability. Cell viability of protopine on HepG2 cells. HepG2 cells were incubated for 24 h with various concentrations 0-50μM protopine. Results are expressed in percentage of non-treated control. Data are expressed as the mean ± SD of three times experiments. $^{**}p < 0.01$ and $^{***}p < 0.001$ compared to non-treated.

Table S1. Antibody used for western blot analysis

Name	Company	Source	Molecular Weight(kDa)	Dilution for WB
β-actin	sc-47778	Mouse	45	1:3000
I-κB	4812S	Rabbit	39	1:1000
p- I-κB	2859S	Rabbit	39	1:1000
NF-κB	8242S	Rabbit	65	1:1000
COX-2	sc-376861	Mouse	72	1:1000
MMP-9	sc-13520	Mouse	92	1:1000
ERK	4695S	Rabbit	42	1:1000
p-ERK	4370S	Rabbit	42	1:1000
JNK	9252S	Rabbit	48	1:1000
p-JNK	4668S	Rabbit	48	1:1000
p38	9212s	Rabbit	40	1:1000
p-p38	4511S	Rabbit	40	1:1000

Table S2. PCR primers used for quantitative real-time PCR

Gene		Sequence (5'~3')	Annealing Temperature(℃)
MMP-9 (Human)	Forward	GCTGCCTGTCGGTGAGATTGG	65.3
MMP-9 (Human)	Reverse	TGGTCCTGGTGCTCCTGGTG	64.6
GAPDH (Human)	Forward	AGCCTTCTCCATGGTCGTGA	60.5
GAPDH (Human)	Reverse	CGGAGTCAACGGATTTGGTC	60.5
